# Case report: internal limiting membrane drape sign masking by foveal detachment in macular telangiectasia type 2

**DOI:** 10.1186/s12886-020-01485-y

**Published:** 2020-06-03

**Authors:** Anmar Abdul-Rahman

**Affiliations:** Department of Ophthalmology, Counties Manukau DHB, Auckland, New Zealand

**Keywords:** Foveal detachment, ILM drape, Macular telangiectasia

## Abstract

**Background:**

Internal limiting membrane (ILM) drape sign is an important OCT characteristic of Macular telangiectasia type 2 (MacTel 2). Described here is a case where masking of the ILM drape sign occurred with bilateral foveal detachments in a patient with MacTel 2.

**Case presentation:**

A 64-year old female was diagnosed with MacTel 2, four years prior to the current presentation on the basis of an OCT demonstrating bilateral ILM drape sign. Fluorescein angiography showed bilateral dilated, ectatic capillaries and late phase dye leak. At the current presentation there was bilateral gradual visual impairment over two months due to bilateral foveal detachments. Treatment with intravitreal Bevacizumab resulted in unmasking of the pre-existing ILM drape sign at 12 weeks. Visual acuity was reduced to counting fingers in the left eye with the neovascular membrane as a consequence of sub-retinal fibrosis, while the right eye maintained a vision of 6/12. A difference in the stage of the disease at presentation determined the long-term visual outcome after seven years of observation.

**Conclusion:**

Foveal detachment can influence the OCT detectability of pre-existing foveal cystoid lesions. Visual prognosis at the final follow up was consistent with the interocular disparity of the disease stage at presentation.

## Background

MacTel 2 is an acquired neurodegenerative and vasculopathic macular disorder [1]. Yanuzzi et al. proposed two distinct types: Type 1 or aneurysmal telangiectasia and the more common Type 2 perifoveal telangiectasia, which can be further classified into a non-proliferative and a proliferative stage [2].

It is well recognised that OCT has allowed the detection of a wide range of foveal signs in MacTel not previously identified with other diagnostic modalities. In a population of 310 patients, Clemons et al. described OCT signs in 74% of eyes, which included apparent foveal detachment, inner/outer photoreceptor segment breaks, hyper-reflectivity or loss of the outer nuclear layer, intra-retinal pigment migration, subretinal neovascularization (SRNV)/fibrosis, and foveal cystoid lesions of the inner retinal layers [3]. In addition to outer retinal layer atrophy, a poor correlation between retinal thickening on OCT and fluorescein angiographic leakage is a recognized feature [2, 4]. The ILM drape sign occurs when a thin membrane overhangs this central cystoid lesion at the base of the fovea of normal contour and thickness. Neither perifoveolar edema nor cystoid spaces outside the focal area are observed on OCT imaging [4].

Presented here is a case of MacTel 2 with bilateral foveal detachments masking a pre-existing ILM drape sign.

## Case presentation

**Fig. 1:**
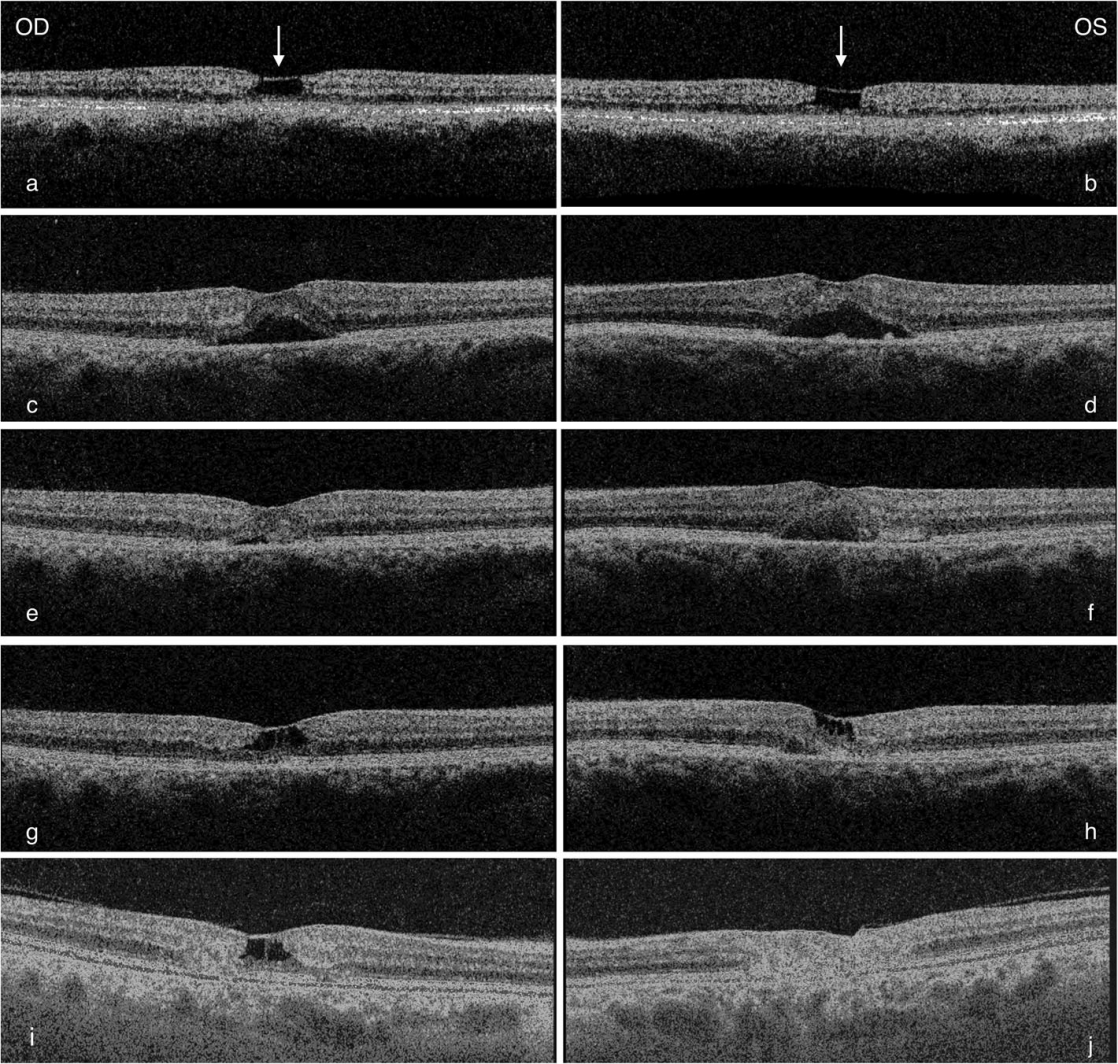
Optical Coherence Tomography (OCT) of the right (**a**, **c**, **e**, **g**, **i**) and left macula (**b**, **d**, **f**, **h**, **j**). **a**, **b** 4-years prior to the current presentation showing foveal cystoid change with internal limiting membrane (ILM) drape (white arrows). **c**-**h** During treatment with six-weekly intravitreal Bevacizumab there was masking of the foveolar cystoid changes previously seen in **a**,**b** and a thickened central fovea (**g**-**h**). Resolution of the subretinal fluid with treatment allowed unmasking of the foveal cystoid changes (**i**, **j**). Final observation at seven years (**i**) outer retinal disruption temporally by a retinal pigment epithelial plaque, persistence of the cystoid change, (**j**) disruption of the retinal architecture by fibrotic change

**Fig. 2:**
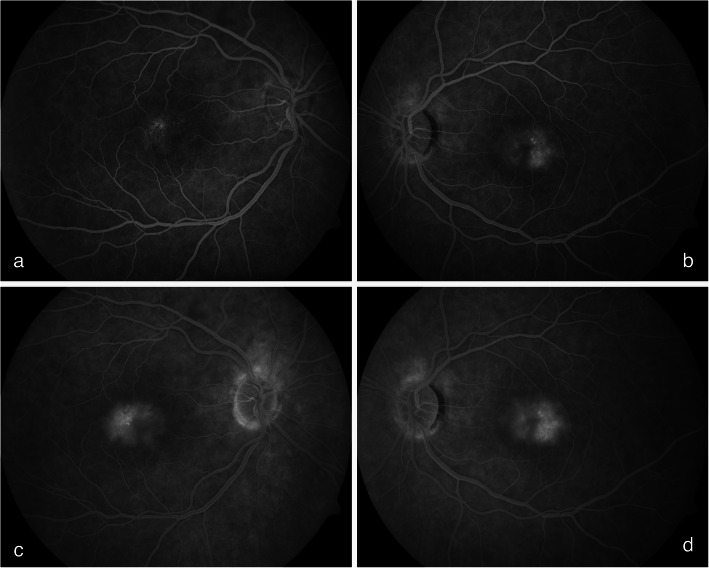
Fluorescein angiogram, clinical signs of sub-retinal neovascularization of the left eye was not detectable clinically at the time of the angiogram one year prior to presentation (**a**, **b**). Mid phase demonstrating dilated, ectatic capillaries **c**,**d** dye leak predominantly in the parafoveal temporal areas seen on the late phase angiogram

A 64-year-old Caucasian female presented with bilateral reduction in vision and metamorphopsia for two months. A presumptive diagnosis of MacTel 2 was established four-years prior to the current presentation on the basis of bilateral temporal macular telangiectatic vessels and OCT imaging exhibiting the ILM drape sign as shown in Fig. 1 (a,b). The diagnosis of MacTel 2 was confirmed on fluorescein angiography, which showed bilateral dilated, ectatic capillaries with late phase dye leak predominantly in the parafoveal temporal areas as shown in Fig. 2 (a-d) one year prior to the current presentation.

**Fig. 3:**
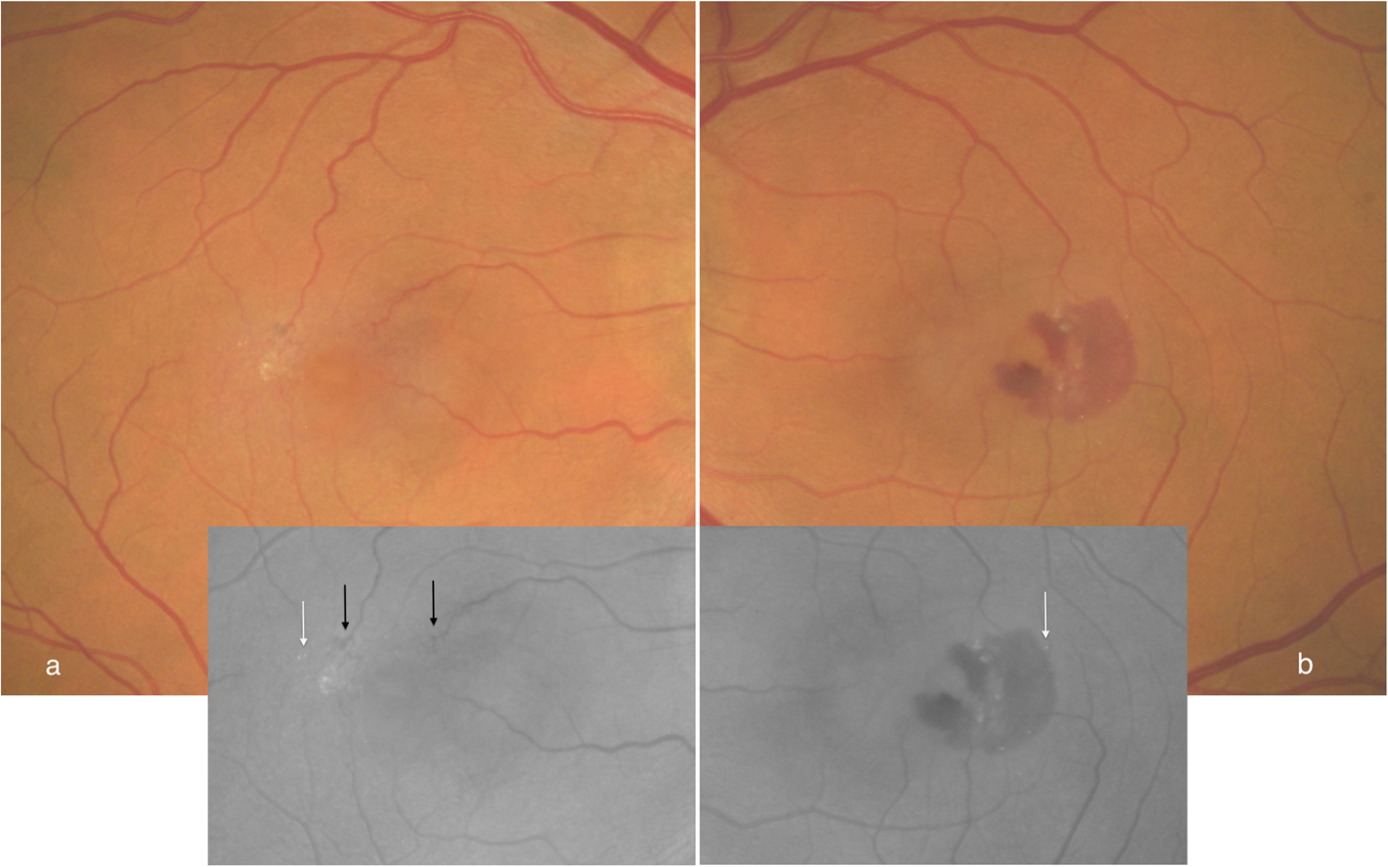
Color fundus photographs. Bilateral loss of foveal details the right macula (**a**) showed retinal opacification temporally with dilated right-angled telangiectatic vessels (inset black arrows). The left eye (**b**) demonstrated a half disc diameter area of subretinal hemorrhage temporally. Bilateral refractile crystalline structures are seen (inset white arrows)

On examination visual acuity was 6/15 and 6/18 in the right and left eyes, respectively. Slit-lamp biomicroscopy of the right macula demonstrated temporal retinal opacification, with dilated right-angled telangiectatic vessels. Refractile crystalline structures were noted in both maculae associated with loss of foveal details. Central macular thickness was 318 μm and 348 μm on the right and left eyes, respectively. There was bilateral asymmetrical sub-retinal fluid consistent with foveal detachments. Although there was disruption of the outer retinal architecture, the foveal contour remained identifiable. Previously noted foveal cystoid changes were undetectable on this occasion as shown in Fig. 1 (c, d). An additional diagnosis of left SRNV was made on the basis of a half a disc diameter area of sub-retinal hemorrhage temporal to the foveola at three weeks follow-up as shown in Fig. 3 (a,b). As a decision was taken not to perform a fluorescein angiogram, treatment was given to the right eye, assuming underlying proliferative change, given the presentation of the left eye, and the disease symmetry on a previous fluorescein angiogram. Four doses of bilateral sequential intravitreal Bevacizumab 1.25 mg/0.1 ml were administered six weeks apart. By 12 weeks after the initial presentation, visual acuity stabilized at 6/12 bilaterally; slit-lamp biomicroscopy demonstrated regression of the sub-retinal hemorrhage in the left eye. Resolution of the foveal detachments with unmasking of the intraretinal foveal cystoid changes are shown on the OCT in Fig. 1 (g-h).

Visual acuity declined to counting fingers in the left eye after three years of follow up as a consequence of sub-retinal fibrosis, the right eye maintained a vision of 6/12. Retinal examination showed an RPE plaque temporal to the macula, resulting in disruption of the temporal retinal architecture Fig. 1 (i, j). These findings remained consistent over a period of observation of seven years and no further intravitreal Bevacizumab was prescribed during this interval.

## Discussion and conclusions

The notable feature in the presented case is the masking of the ILM drape sign in the presence of the foveal detachments. The limitation of this case report was the absence of a repeat fluorescein angiogram at the second presentation. However, the diagnosis was established by the weight of the clinical evidence and the previous angiogram results, therefore, the need for further invasive testing was difficult to justify.

First described by Gass and Blodi as pseudo-lamellar macular holes, cavitatory foveal change was reported in 14 of their series of 184 eyes [5]. Foveal detachment is an uncommon finding in MacTel 2. Mehta et al. reported a 1.4% prevalence in a sretrospective case series of 427 patients. As patients with proliferative changes were excluded from the Mehta et al. study, the prevalence of SRNV in the presence of a foveal detachment was not established [6]. The most common OCT findings in this disease include intraretinal cystoid spaces without foveal thickening, detected in 53% of the 310 participants of the MacTel Project group [3]. This case demonstrates masking of the cavitatory lesion in the presence of intra or sub-retinal fluid, which may further assist to characterize this lesion. It is possible that the hydrostatic force generated by the subretinal fluid, in addition to the intraretinal edema causing collapse of the intraretinal cavity, could have contributed to this finding.

In summary foveal detachment can influence the OCT detectability of pre-existing foveal cystoid lesions. Visual prognosis at the final follow up was consistent with the interocular disparity of the disease stage at presentation.

### Patient perspective

The patient was content with the stabilisation of vision in the right eye, which has allowed her to continue with activities of daily living over the years.

## Data Availability

All data generated analysed during this study are included in this published article.

## Abbreviations

ILM: Internal limiting membrane
Mac Tel: Macular telangiectasia
OCT: Optical coherence tomography
SRNV: Subretinal neovasclarization
RPE: Retinal pigment epithelium
